# p75 neurotrophin receptor preserves neuromuscular synapse stability and muscle strength during aging

**DOI:** 10.1038/s41514-026-00399-1

**Published:** 2026-05-16

**Authors:** Viviana Pérez, Sebastián Aedo-Cares, Francisca Bermedo-García, Jessica Mella, Camila Uribe-Martínez, Francisca C. Bronfman, Juan Pablo Henríquez

**Affiliations:** 1 Departamento de Fisiología, Facultad de Ciencias Biológicas, Universidad de Concepción, Concepción, Chile; 2 Departamento de Biología Celular, Facultad de Ciencias Biológicas, Universidad de Concepción, Concepción, Chile; 3 Neuromuscular Studies Lab (NeSt Lab), Instituto de Anatomía, Histología y Patología, Facultad de Medicina, Universidad Austral de Chile, Valdivia, Chile; 4 Institute of Biomedical Sciences (ICB), Faculty of Medicine, and Faculty of Life Sciences, Universidad Andrés Bello, Santiago, Chile

**Keywords:** Neuroscience, Physiology

## Abstract

Age-related decline of the neuromuscular junction (NMJ), the peripheral synapse that controls muscle contraction, contributes to muscle weakness and impaired motor function in aging. The NMJ comprises a motor axon terminal, a skeletal muscle fibre, and terminal Schwann cells (tSC). Neurotrophin signalling is essential for mature NMJ organisation, with the p75 receptor acting as a key regulator of its morphology and function. However, the potential contribution of p75 to age-related NMJ decline remains unexplored. In this study, we used germline p75 knockout (p75⁻/⁻) mice to examine how the lifelong absence of p75 impacts NMJ stability and muscle function during aging through quantitative morphometric analyses, postsynaptic receptor dynamics, muscle histology, and functional strength testing. Although NMJ morphology was preserved, aged p75⁻/⁻ mice exhibited pronounced denervation and reduced tSC coverage, hallmarks of age-associated NMJ degeneration. Moreover, postsynaptic domains of aged p75⁻/⁻ mice displayed reduced stability of membrane-bound acetylcholine receptors. Glycolytic muscle fibres also showed signs of atrophy. Notably, aged p75⁻/⁻ mice exhibited a significant reduction in muscle strength compared with age-matched controls. Together, our findings are consistent with cumulative effects of persistent p75 deficiency on NMJ integrity and function during aging, supporting its potential relevance for interventions aimed at preventing age-associated neuromuscular decline.

## Introduction

The neuromuscular junction (NMJ) is a peripheral cholinergic synapse formed by the axon terminal of a motor neuron, a skeletal muscle fibre region enriched in acetylcholine receptors (AChR), and capping terminal Schwann cells (tSC)^1,2^. Proper formation, maturation, and maintenance of the NMJ requires coordinated signalling among its three components to effectively fulfil its physiological role of initiating muscle contraction. Various conditions, including motor diseases, traumatic injury, and aging, disrupt the precise organization and stability of the three cellular partners of the NMJ, leading to functional impairment^3,4^.

Aging involves the progressive deterioration of cellular interactions, tissue structures, and function over time. In murine species, NMJ disorganisation becomes evident from approximately 18 months of age and is characterised by postsynaptic alterations, including fragmentation of AChR aggregates, endplate area expansion, and aberrant spreading towards extrasynaptic regions of the sarcolemma (i.e. outside the AChR-labelled endplate)^5,6^. In parallel, presynaptic axons become thinner, display escape fibre extensions, and poly-innervate postsynaptic domains^5,6^; meanwhile, tSCs become smaller and progressively detach from the NMJ^7^. As a result of morphological decline, later aging stages exhibit motor axon retractions resulting in partial to complete denervation^8^. Interestingly, NMJs in different skeletal muscles show marked variability in their susceptibility to aging, ranging from highly vulnerable to largely age resistant, with some muscles affected only at late stages of life. This heterogeneity is likely related to the anatomical distribution of muscles and their patterns of innervation^9^. Despite the detailed morphological characterization of its three cellular components, the molecular determinants of age-dependent NMJ decline remain largely unknown.

The neurotrophin (NT) family of growth factors plays diverse functions in the nervous system by binding and activating specific tyrosine kinase receptors (Trks)^10^. Specifically, the effects of neurotrophins on neuronal survival and growth depend on the binding of the nerve growth factor (NGF) to TrkA, the brain-derived neurotrophic factor (BDNF) and NT-4 to TrkB, and NT-3 to TrkC^10–12^. Additionally, NTs and their precursor forms (pro-NTs) bind to p75, a multifunctional signalling receptor belonging to the tumour necrosis factor receptor family^10,13^.

Although p75 expression is downregulated with adulthood^14^, all three components of the NMJ maintain low levels of p75 expression^15,16^. The p75 receptor is strongly re-expressed in motor neurons and Schwann cells in response to nerve injury or in models of the motor disease amyotrophic lateral sclerosis^17–21^. At the NMJ, p75 signaling contributes to the mechanisms regulating perinatal motor axon pruning of poly-innervated NMJs and acetylcholine (ACh) release^16,22–24^. The p75 receptor is also required for postsynaptic maturation and stability at the adult NMJ, likely controlling the number of presynaptic vesicles in motor axon terminals^25^. Consequently, p75 knockout (p75⁻/⁻) mice exhibit muscle weakness, decreased muscle strength, and alterations in motor coordination^25,26^. Besides the evidence connecting p75 with mature NMJ maintenance, a possible role for p75 at the aged NMJ has not been explored.

In this study, we conducted comprehensive morphometric analyses of the NMJ and muscle in aged mice lacking p75 throughout life (p75⁻/⁻)^26^. Interestingly, some phenotypes of p75 silencing observed at the NMJ of adult animals have been compensated in aged muscles, including most pre- and postsynaptic phenotypes, such as postsynaptic fragmentation. However, aged p75⁻/⁻ NMJs display impaired stability of AChRs in the muscle membrane, concomitant with denervation of postsynaptic domains. We also found that aged p75⁻/⁻ mice display muscle weakness compared to aged controls. Together, our findings reveal that persistent p75 deficiency compromises NMJ stability and muscle function during aging.

## Results

### The p75 neurotrophin receptor is expressed in muscle and at the NMJ during aging

**Fig. 1:**
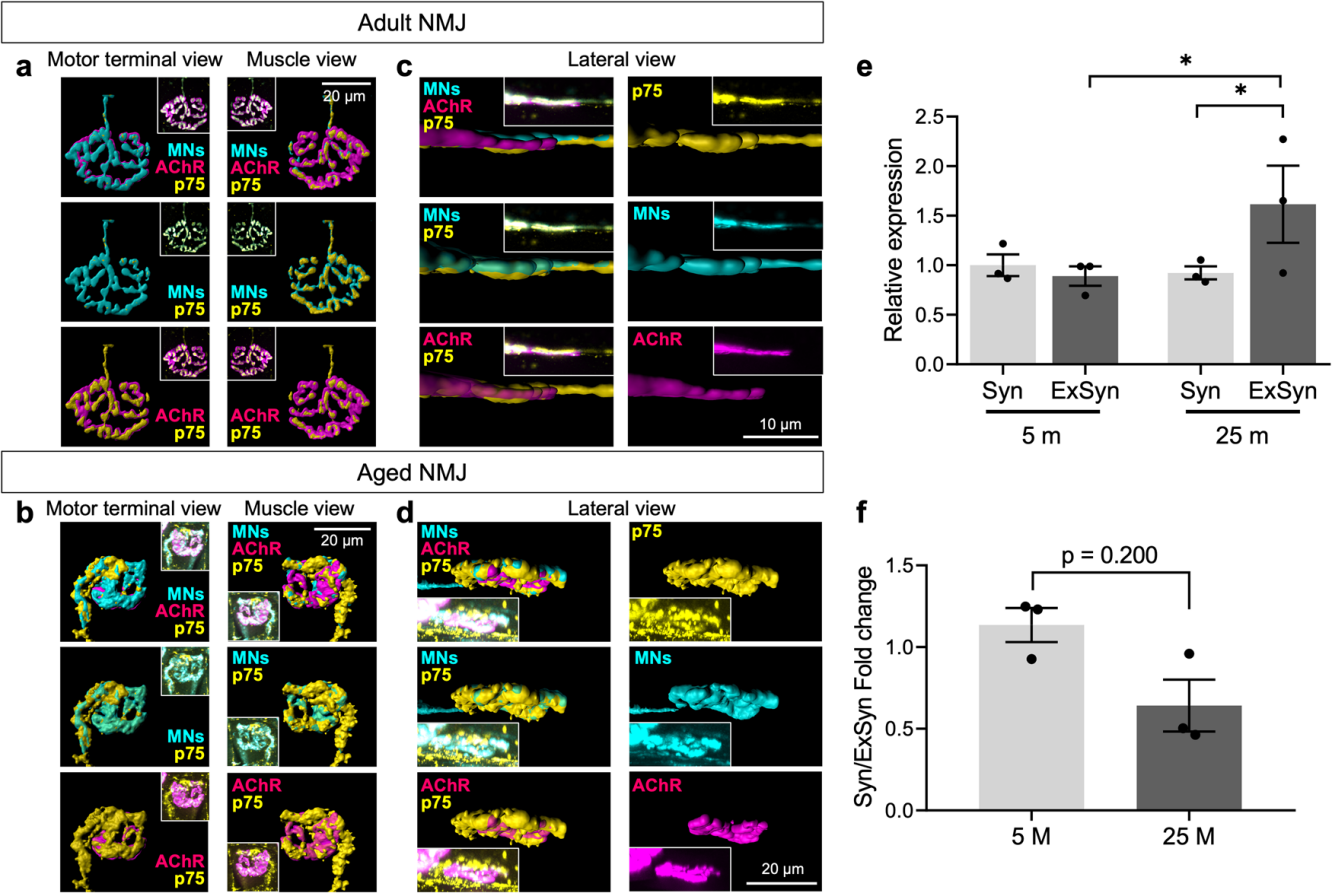
p75 is expressed at the aged NMJ. Whole-mount lumbrical muscles from control adult (5-mo-old) (**a**, **c**) and aged (24–26-mo-old) (**b**, **d**) mice were immunostained to reveal the motor axon terminal (2H3 + SV2 antibodies; cyan), AChRs (BTX; magenta), and p75 (yellow). Images acquired as z-stacks were 3D reconstructed using IMARIS software. Left panels show the 3D motor terminal view and the corresponding rotated muscle view of representative NMJs (**a**, **b**). The right panels show a 3D reconstructed lateral view of a representative NMJ region. Insets show non-3D-processed immunostainings. **e** p75 mRNA expression quantification relative to GAPDH in synaptic (Syn) and extrasynaptic (ExSyn) regions of the diaphragm from adult and aged mice. **f** Relative p75 mRNA expression in synaptic vs. extrasynaptic regions from adult and aged mice. Results are shown as mean ± SEM (bars) with individual values (dots; *N* = 3–4 mice). One-way ANOVA (**e**) and Mann-Whitney test (**f**) were performed. n.s. = non-significant. Scale bar = 20 µm (**a**, **b**, **d**) and 10 µm (**c**).

To study a possible involvement of the p75 receptor on the maintenance of the aged NMJ, we analysed the expression profile of this neurotrophin receptor in skeletal muscle samples from adult (5-mo-old) and aged (25-mo-old) mice (Fig. 1). Immunohistochemical experiments were performed using a specific anti-p75 antibody (Fig. 1a–d; yellow) along with NMJ markers in the distal hindlimb lumbrical muscles, the ones controlling phalangeal flexion. NMJs were labelled using anti-neurofilaments plus anti-synaptic vesicles antibodies to reveal presynaptic motor terminals (Fig. 1a–d; cyan) and with BTX (Fig. 1a–d; magenta) to stain AChRs located in postsynaptic densities. To analyse the colocalisation of p75 with both (Fig. 1a, b; upper panels) or with each different cell type of the NMJ (Fig. 1a, b; middle and lower panels), 3D reconstructions were obtained from confocal z-stacks and oriented to visualise the motor terminal view (Fig. 1a, b; left panels) or rotated to reveal the muscle view (Fig. 1a, b; right panels). Insets show the unprocessed maximum intensity projection (MIP) image of the z-stacks from each NMJ. According to previous evidence showing that p75 is expressed in the three cell partners of the NMJ^15,16^, muscles from adult mice imaged in the *in face* orientation show an evident staining for p75 at the NMJ level (Fig. 1a; top panels). Interestingly, despite a punctate distribution, p75 shows a similar localization at the aged NMJ (Fig. 1b; top panels). The p75 receptor is detected in the motor axon portion contacting the endplate as well as in the motor terminal covering the endplate (Fig. 1a, b; middle panels). In addition, p75 also displays a clear distribution within the postsynaptic domain in adult and aged mice (Fig. 1a, b; bottom panels). In a lateral view, p75 staining was compared with the two (Fig. 1c, d; left upper panels) or with each different cell type of the NMJ (Fig. 1c, d, left middle and lower panels). Single detections are also displayed (Fig. 1c, d, right panels). Our analyses confirm that p75 staining covers the entire NMJ area, co-distributing with both the presynaptic (Fig. 1c, d; left middle panels) and the postsynaptic (Fig. 1c, d; left bottom panels) domains of adult and aged NMJs.

Next, to analyse the level of expression of p75 in muscles, receptor transcripts were amplified from samples obtained from synaptic (i.e. containing NMJs) and extrasynaptic regions of diaphragm muscle fibres. RT-qPCR analysis shows that p75 is expressed in both muscle fibre domains. Interestingly, while p75 expression in the innervated muscle region was not altered with aging, its expression is significantly up-regulated in the muscle extrasynaptic domain in aged as compared to adult mice (0.89 ± 0.09 in 5-mo-old and 1.62 ± 0.39 in 25-mo-old) (Fig. 1e). Indeed, the ratio of p75 expression levels between both muscle domains is almost 2-fold higher in adult vs aged mice (Fig. 1f). Together, these findings show that p75 is expressed at the NMJ of aged mice and that its expression is specifically upregulated in the muscle extrasynaptic region.

### The p75 receptor is dispensable for presynaptic morphology but required for terminal Schwann cell coverage during aging

**Fig. 2:**
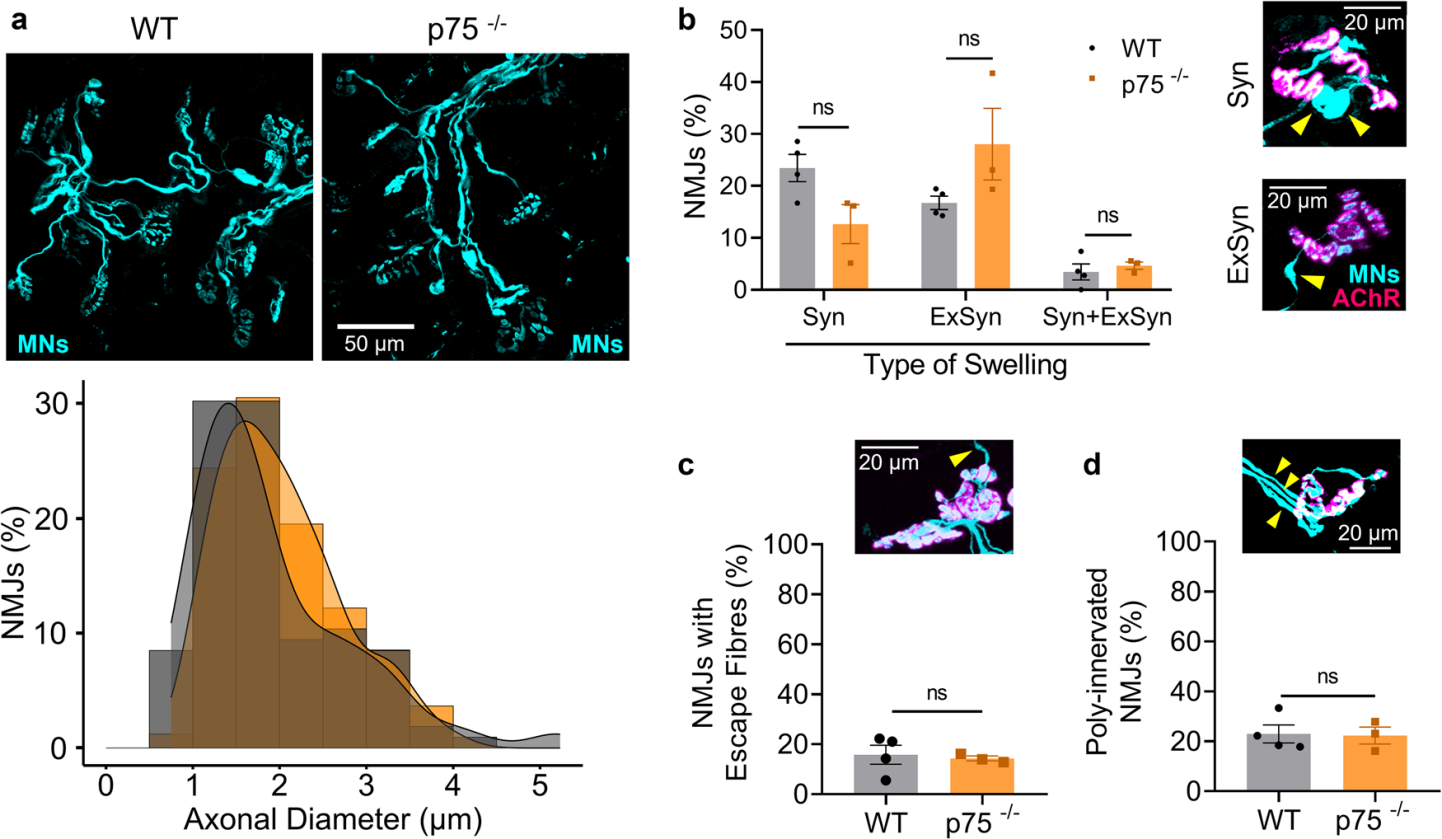
p75⁻/⁻ mice show no alterations in the presynaptic motor terminal of the NMJ during aging. Whole-mount lumbrical muscles from aged control and p75⁻/⁻ mice were immunostained to reveal motor axons and presynaptic terminals (2H3 + SV2 antibodies; cyan) and postsynaptic AChRs (BTX; magenta). **a** Upper panels show representative images of motor axon terminals from aged control and p75⁻/⁻ mice; the lower panel shows a histogram of axonal diameter distribution. Scale bar = 50 µm. **b** Quantification of the percentage of NMJs with presynaptic swelling in synaptic (Syn; arrowheads in top panel) or extrasynaptic (ExSyn; arrowhead in bottom panel) regions in aged control and p75⁻/⁻ mice. **c** Proportion of NMJs with escape fibres (arrowhead in image) in aged control and p75⁻/⁻ mice. **d** Percentage of poly-innervated NMJs (arrowheads in image) in aged control and p75⁻/⁻ mice. Scale bar = 20 µm (**b**–**d**). Data are shown as mean ± SEM (bars) and individual values (dots; *N* = 3–4 mice). Two-way ANOVA (**a**, **b**) and Mann-Whitney test (**c**, **d**). n.s. = non-significant.

As p75 is expressed in the three cellular components of the NMJ^15^, next we analysed synaptic morphology in hindlimb lumbrical muscles from aged p75⁻/⁻ and control mice (Fig. 2). We used confocal laser microscopy to first examine presynaptic features typically found in conditions of NMJ decline, such as pathology, nerve damage, or aging^9,27–29^. Confocal image analyses showed no differences between aged control and p75⁻/⁻ mice in presynaptic parameters, such as axonal swelling, escape fibres, or poly-innervation of postsynaptic domains (Fig. 2a–d). A slight but not statistically significant shift to bigger axonal diameter was quantified in the aged p75⁻/⁻ mice (Fig. 2a, lower panel). Together, these findings suggest that age-related alterations of presynaptic parameters are mainly independent of p75 expression.

**Fig. 3:**
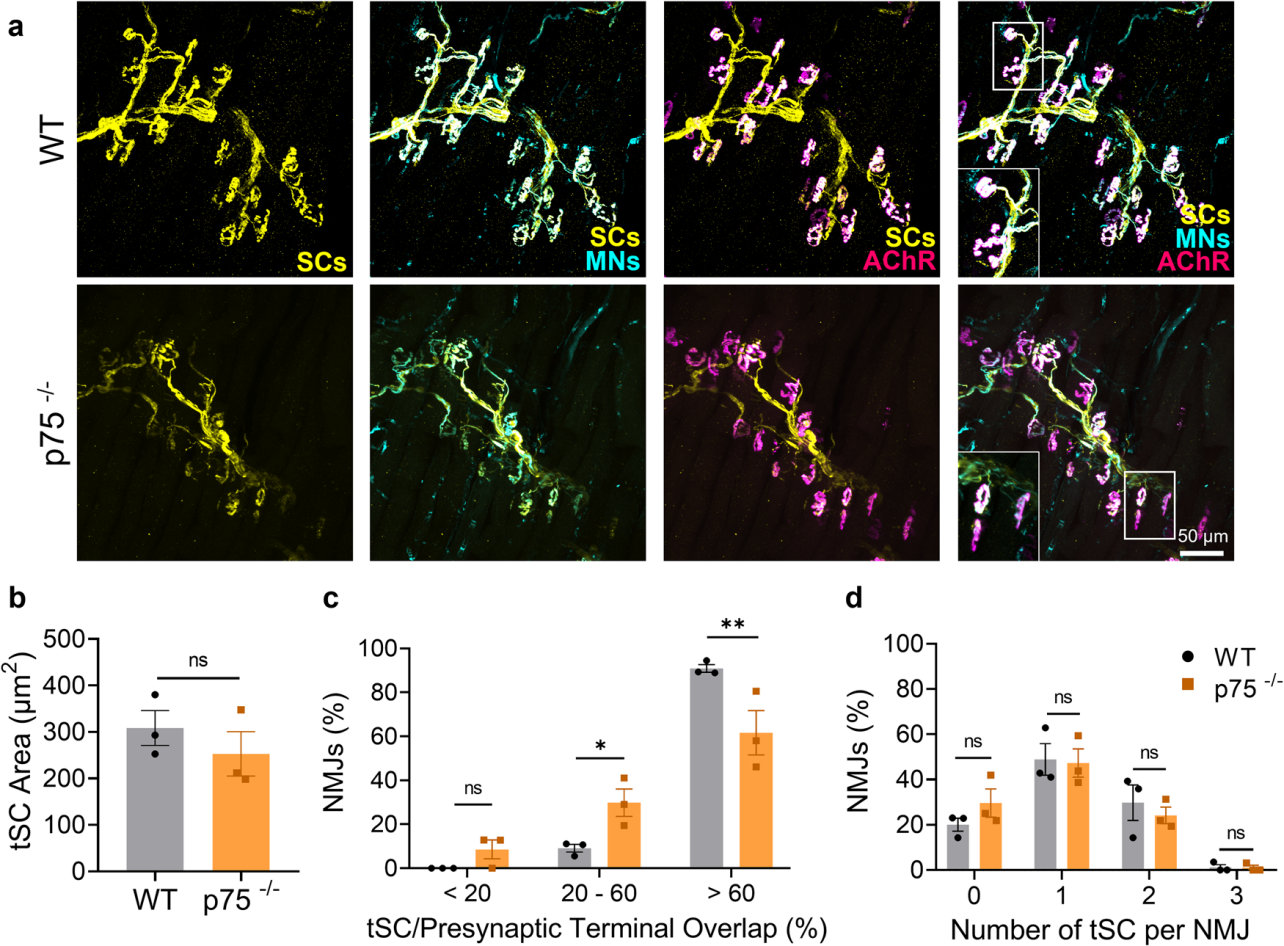
Aged p75⁻/⁻ mice exhibit impaired tSC coverage at the NMJ. **a** Lumbrical muscles from aged control and p75⁻/⁻ mice were stained for motor axons (2H3 + SV2 antibodies; cyan), tSCs (S100β; yellow), and postsynaptic AChRs (BTX; magenta). Scale bar = 50 μm. Quantification of tSC area (**b**) percentage of tSC overlap with presynaptic terminals (**c**), and number of tSCs per NMJ (**d**) in aged control and p75⁻/⁻ mice. Data are shown as mean ± SEM (bars) and individual values (dots; *N* = 3–4 mice). *p < 0.05, **p < 0.01, Mann-Whitney test (**b**) two-way ANOVA (**c**, **d**). n.s. = non-significant.

The expression of p75 in Schwann cells at perinatal stages controls NMJ poly-innervation, while in adulthood tSC-derived p75 is up-regulated upon nerve damage^14,15,30,31^. To analyse the behaviour of tSCs in aged muscles from control and p75⁻/⁻ mice, we analysed immunohistochemical staining using the anti S100β antibody alone (Fig. 3a, fist column), or along with each different cell type of the NMJ (Fig. 3a, second and third columns), or the three of them together (Fig. 3a, fourth column). Quantitative analyses revealed that although tSCs area was unaffected (Fig. 3b), aged muscles from p75⁻/⁻ mice display a reduced overlapping area of Schwann cells on presynaptic motor axon terminals (Fig. 3c). Indeed, distribution analyses of tSC coverage of NMJs shows that, compared to controls, p75⁻/⁻ mice exhibit a significant increase of low ( < 20um^2^= zero in controls to 8.58 ± 4.29% in p75⁻/⁻ mice); and middle coverage (20-60 um^2^ = 9.13 ± 1.79% in controls to 29.83 ± 6.24% in p75⁻/⁻ mice), along with a concomitant decrease of higher overlapping areas ( > 60um^2^ = 90,87 ± 1,79% in controls to 61.60 ± 10.09 in p75⁻/⁻ mice) (Fig. 3c). To distinguish if the reduced capping of tSCs was due to the retraction of cell projections or to cell detaching, the number of Schwann cell nuclei per NMJ was quantified. Our results show no significant differences between p75⁻/⁻ and control mice (Fig. 3d). Our findings reveal that tSC projections covering the NMJ are reduced in muscles from p75⁻/⁻ mice during aging.

### The p75 neurotrophin receptor is required for postsynaptic AChR stability, but not morphology, at the aged NMJ

**Fig. 4:**
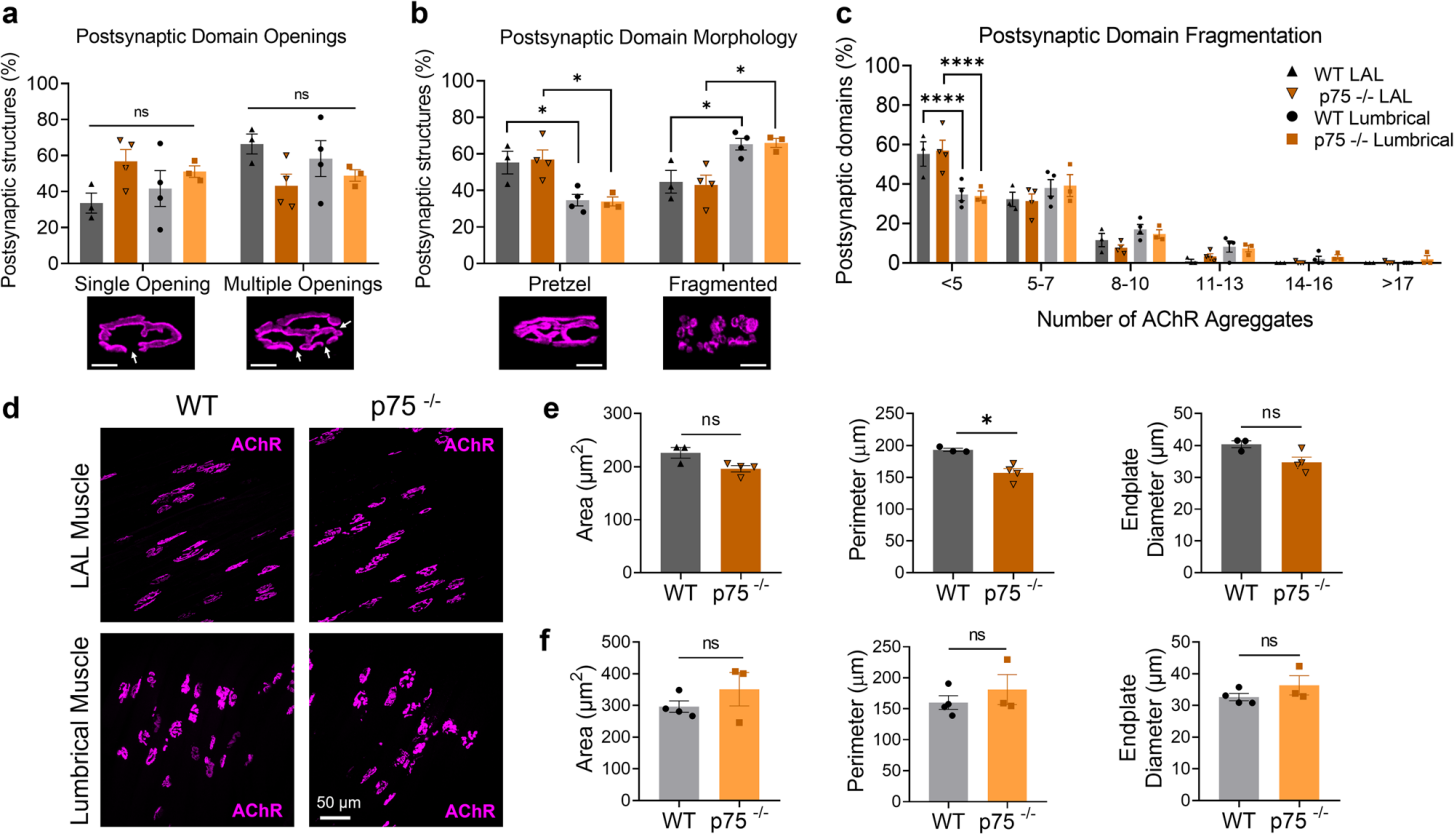
The NMJ postsynaptic structure is unaltered in aged p75⁻/⁻ mice. Whole-mount proximal (LAL; dark gray and orange) and distal (lumbrical; light gray and orange) muscles from aged control and p75⁻/⁻ mice were stained to reveal NMJ postsynaptic densities (BTX; magenta). **a** Postsynaptic domains were categorised based on complexity as having single openings (less mature) or multiple openings (more mature), and quantified. NMJ fragmentation was evaluated as the percentage of fragmented structures (**b**) and by the distribution in the number of AChR aggregates (**c**). **d** Representative confocal images of postsynaptic structures in LAL (top) and lumbrical (bottom) muscles. Scale bar = 50 µm. Quantification of area, perimeter, and endplate diameter in LAL (**e**) and lumbrical (**f**) muscles. Results are shown as mean ± SEM (bars) and individual values (dots; *N* = 3–4 mice). *p < 0.05, **p < 0.01, two-way ANOVA (**a**–**c**), Mann-Whitney test (**e**, **f**). n.s. = non-significant.

Based on previous findings showing that p75⁻/⁻ mice delay postsynaptic maturation in adult LAL muscles^25^, we aimed to determine the potential contribution of p75 expression to maintain postsynaptic NMJ morphology in aged muscles (Fig. 4). Considering the susceptibility of different muscles to aging^9^, in these studies we compared the LAL (proximal) and lumbrical (distal) muscles. As we previously reported^25^, AChR aggregates were categorised into single opening (less mature) or multiple opening pretzels (full mature) (Fig. 4a), which were subsequently subdivided into fragmented or mature pretzels (Fig. 4b). In fragmented NMJs, we also determined the number of AChR aggregates in both conditions (Fig. 4c). Although we observed that NMJs from lumbrical muscles were more fragmented that those from the LAL muscle (53,95 ± 7,61% and 49.81 ± 5.68% of postsynaptic domains with less than five fragments in control and p75⁻/⁻ LAL muscles to 35.45 ± 6.69% and 31.64 ± 4.02% in control and p75⁻/⁻ lumbrical muscles) (Fig. 4c), we found no differences in the morphology of postsynaptic apparatuses in the NMJ of aged p75⁻/⁻ mice compared to control mice in both muscles (Fig. 4a–c). Consistent with these findings, quantification of postsynaptic organisation parameters such as area, perimeter, and diameter, showed no differences between control and p75⁻/⁻ mice, except for a slight but significant decrease in the pretzel perimeter specifically in the LAL muscle of p75⁻/⁻ mice (193.5 ± 2.48 µm) compared with controls (157.2 ± 6.91 µm) (Fig. 4e). Thus, the absence of p75 results in no significant alterations of the morphology of NMJ postsynaptic structures in aged proximal and distal muscles.

**Fig. 5:**
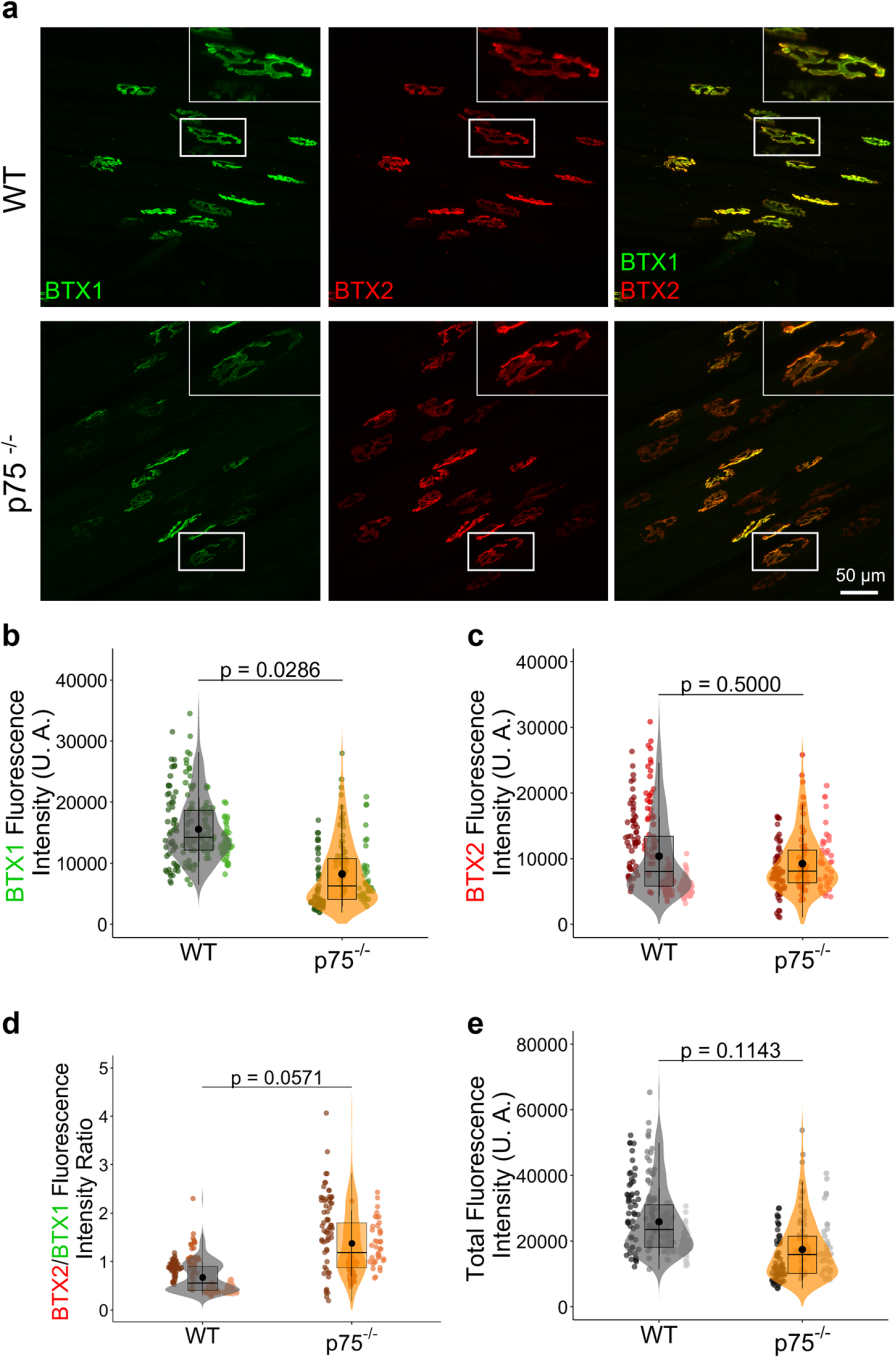
p75 inhibition destabilises the aged postsynaptic domain at the NMJ. Aged control and p75⁻/⁻ mice were injected subcutaneously in the head/neck region with Alexa-488-conjugated BTX (green; BTX1). After 7 days, LAL muscles were dissected and stained with Alexa-555 BTX (red; BTX2). Changes in BTX1 fluorescence intensity reflect alterations in pre-existing, membrane-bound AChRs, whereas BTX2 labeling reflects the insertion of newly synthesised receptors. **a** Representative confocal images of NMJ postsynaptic domains. Scale bar = 50 µm. Quantification of BTX1 (**b**) BTX2 (**c**) fluorescence intensity, BTX2/BTX1 ratio (**d**) and total staining intensity (BTX1 + BTX2) (**e**). Data are shown as violin plots with individual NMJs overlaid (dots) and per-animal values (columns; *N* = 3–4 mice, 38–63 NMJs per mouse). Statistical analyses were performed using animal means; individual NMJs are shown to illustrate within-animal variability. *p* values were estimated using Mann–Whitney test (**b**–**e**).

Although in adult LAL muscles, AChR stability is not altered by the absence of p75 expression^25^, several conditions resulting in NMJ decline correlate with reduced AChR stability in the muscle membrane^32,33^. Therefore, we next aim to analyse postsynaptic density and stability using the two-colour BTX assay in aged LAL muscles from both genotypes (Fig. 5). For this, muscles from aged control and p75⁻/⁻ mice were stained in vivo with an unsaturating concentration of BTX conjugated to a first fluorophore (BTX1) and after 7 days, newly inserted AChRs into the membrane were stained with a second fluorescently conjugated BTX (BTX2)^34^ (Fig. 5a). Quantification of BTX fluorescence intensities shows that the population of pre-existing surface AChRs is significantly reduced in the NMJs of p75⁻/⁻ LAL muscle (8,546 ± 1,880 U.A. in p75⁻/⁻ mice to 15,544 ± 797 U.A. in controls) (Fig. 5b), while the proportion of newly incorporated receptors was unaffected (Fig. 5c). Consequently, the proportion of BTX2 fluorescence intensity related to BTX1 is augmented upon p75 silencing (1.33 ± 0.25) compared to controls (0.65 ± 0.25), consistent with reduced stability and increased loss of pre-existing AChRs in the muscle membrane of p75⁻/⁻ mice (Fig. 5d). Instead, the total density of AChRs, estimated by the total (BTX1 + BTX2) fluorescence intensity, was not significantly altered by the absence of p75 expression in the aged LAL muscle (Fig. 5e). Together, these findings show that the chronic inhibition of p75 expression results in reduced stability of postsynaptic AChRs in aged muscles.

### The p75⁻/⁻ mice display increased age-related NMJ denervation and muscle weakness

**Fig. 6:**
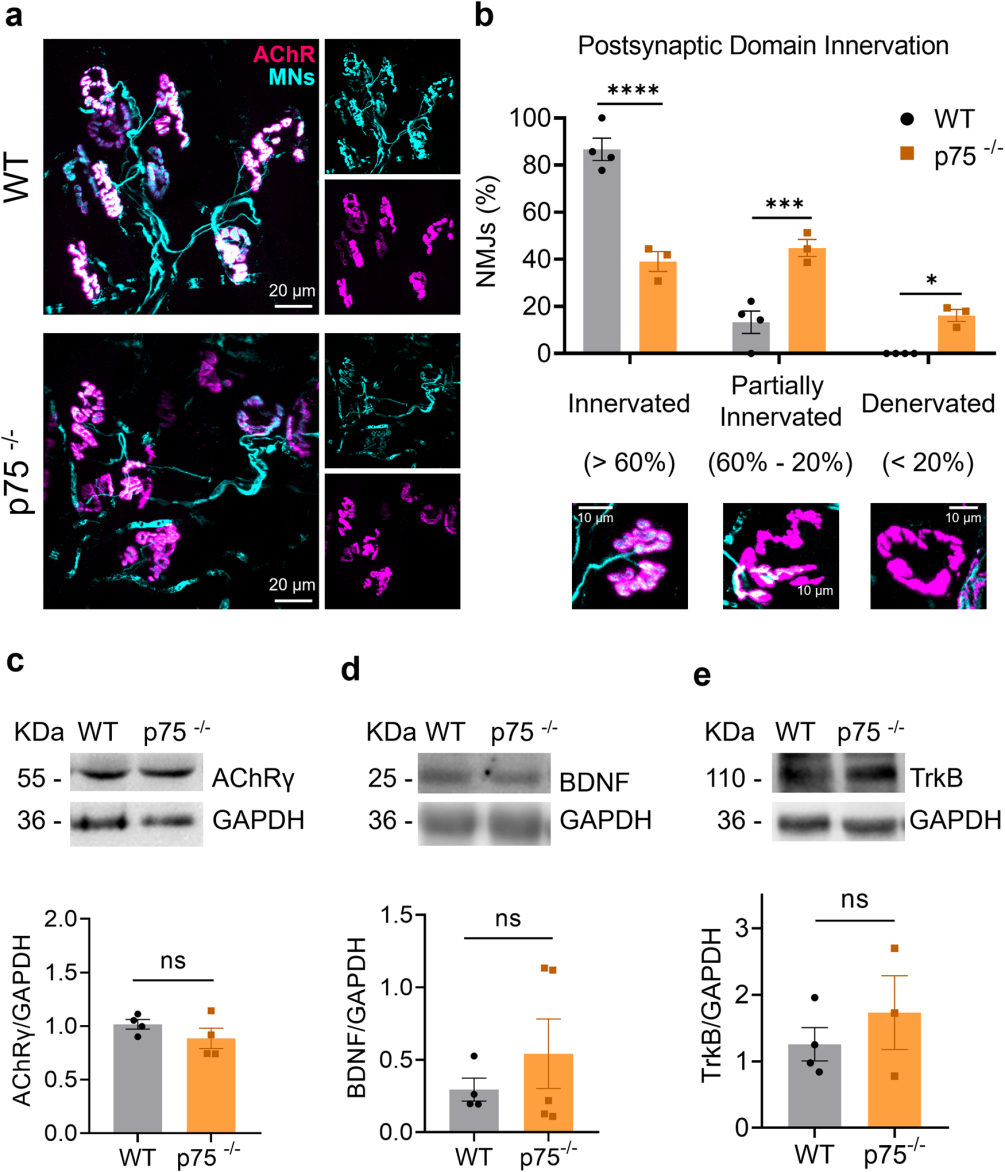
p75⁻/⁻ mice show enhanced NMJ denervation during aging. Whole-mount lumbrical muscles from aged control and p75⁻/⁻ mice were stained to reveal presynaptic axons (2H3 + SV2 antibodies; cyan) and postsynaptic AChRs (BTX; magenta). **a** Representative confocal image of NMJs. Scale bar = 20 μm. **b** NMJ innervation was quantified by the overlap between pre- and postsynaptic areas. Bottom panels show examples of innervated ( > 60%), partially innervated (20–60%), and denervated ( < 20%) NMJs. *N* = 3-4 mice. Scale bar = 10 μm. Western blot of Gastrocnemius muscle samples from aged control and p75⁻/⁻ mice (*N* = 3-5 mice) for the AChR γ-subunit (**c**) BDNF (**d**) and TrkB (**e**). GAPDH served as a loading control. Data are shown as mean ± SEM (bars) and individual values (dots). *p < 0.05, ***p < 0.001, ****p < 0.0001, ns non-significant; Two-way ANOVA (**b**) or Mann-Whitney (**c**–**e**) tests.

Considering that aging results in NMJ denervation^6,35,36^ and functional loss^37,38^, we next evaluated NMJ innervation in aged p75⁻/⁻ mice. On confocal images of NMJ staining (Fig. 6a), we quantified the apposition between the pre and postsynaptic domains (Fig. 6b). Based on previous findings^27,39–41^, we considered innervated NMJs as those having >60% presynaptic coverage of postsynaptic domains, NMJs having <20% of coverage were considered denervated, while intermediate apposition was quantified as partially innervated (Fig. 6b). Our findings show that the proportion of fully innervated NMJs significantly decreased from 86.71 ± 4.73% in controls to 39.05 ± 4.20% in p75⁻/⁻ mice, while a similar proportion of NMJs appeared as partially innervated (Ctrl= 13.29 ± 4.73; p75⁻/⁻= 44.81 ± 3.63). Accordingly, the proportion of fully denervated NMJs in lumbrical muscles was significantly augmented from zero in control muscles to 16.14 ± 2.55% in p75⁻/⁻ mice (Fig. 6b). As an initial approach to examine molecular readouts commonly associated with NMJ decline, we considered that muscle denervation upregulates the expression of the fetal γ-AChR subunit^42^). We also considered that neuromuscular activity regulates BDNF and TrkB expression^43^. Western blot analyses of whole-muscle lysates revealed no significant differences in AChR γ-subunit levels between genotypes (Fig. 6c and Supplementary Fig. S1). Similarly, protein levels of BDNF and its TrkB receptor were comparable between p75⁻/⁻ and control muscles (Fig. 6d, e and Supplementary Figs. S2, S3).

**Fig. 7:**
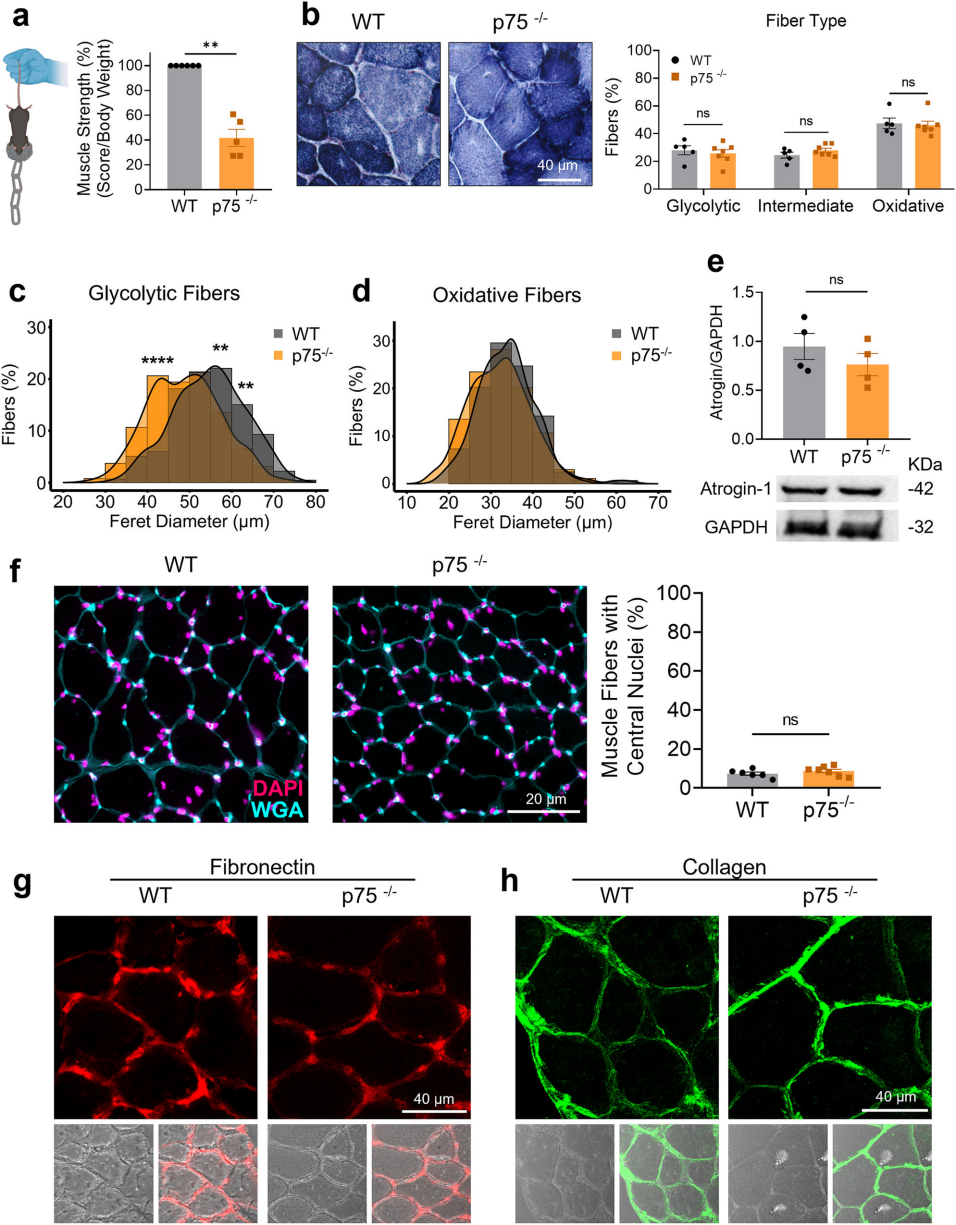
Aged p75⁻/⁻ mice show increased muscle weakness. **a** Aged control and p75⁻/⁻ mice were tested for forelimb strength using a weight-holding task. Muscle strength is expressed relative to holding time and body weight (*N* = 6 control, 5 p75⁻/⁻ mice). **b** TA muscles were sectioned and stained for NADH-TR activity (left panels). Scale bar = 40 μm. Quantification of glycolytic (dark blue; NADH-TR + ), intermediate, and oxidative (light blue; NADH-TR − ) fibres as a percentage of total fibres ( > 400) from a central region of interest of TA muscles (right panel). *N* = 3. Feret’s diameter of glycolytic (**c**) and oxidative (**d**) fibres ( > 100 per genotype) was measured. **e** Western blot of Gastrocnemius muscle samples from aged control and p75⁻/⁻ mice (*N* = 4) for atrogin-1; GAPDH served as a loading control. **f** Cross-sections of TA muscles were stained for WGA and DAPI (left panels). Scale bar = 20 μm, The percentage of fibres with central nuclei was quantified (right panel). *N* = 3. Cross-sections were stained to detect fibronectin (**g**) and collagen (**h**; via second harmonic generation). Lower panels show separate brightfield and fluorescent channels (Scale bar = 40 μm). Data represent mean ± SEM. n.s. = non-significant, **p < 0.01; ****p < 0.0001, Mann-Whitney test (**a**, **e**, **f**), two-way ANOVA (**b**–**d**).

Finally, we analysed the potential impact of p75 inhibition on muscle organisation and function. When subjected to a weight test, aged p75⁻/⁻ animals displayed a significant reduction in their strength performance (41.75 ± 6.92%) compared to aged control mice (100 ± 0.0%) (Fig. 7a). Then, to analyse muscle organisation, transversal cryosections of TA muscles from both genotypes were tested for various parameters (Fig. 7b–d, f–h). We found that while there were no differences in fibre type composition between control and p75⁻/⁻ mice (Fig. 7b), glycolytic fibres displayed significantly reduced cross-sectional area in p75⁻/⁻ mice compared to controls (Fig. 7c, d). Interestingly, no significant differences were found between aged control and p75⁻/⁻ mice in the expression of the muscle atrophy protein Atrogin-1 (Fig. 7e and Supplementary Fig. S4), in the proportion of centrally nucleated fibres, indicative of degeneration/regeneration cycles (Fig. 7f), or in the detection of inter-fibre fibronectin (Fig. 7g) or collagen (Fig. 7h) deposition, as markers of muscle fibrosis.

Altogether, our results show that p75 is expressed at the NMJ during aging and contributes to postsynaptic stability at the muscle membrane and to the organisation of tSCs. Chronic inhibition of p75 is associated with increased NMJ denervation and impaired muscle strength in aged muscles. These findings imply a novel role for the p75 neurotrophin receptor in the structural maintenance of neuromuscular synapses during aging, helping to preserve synaptic connectivity and muscle function.

## Discussion

Our previous work demonstrated that p75 is required to stabilise the mature NMJ, influencing both pre- and postsynaptic organisation and synaptic function in adult mice^25^. Based on these findings, here we examined whether the lifelong absence of this receptor exacerbates structural and functional changes that characterise the aging NMJ. During early postnatal stages, p75 localises to all three cellular components of the synapse and regulates axonal pruning of poly-innervated NMJs^16,44^. In adulthood, receptor levels become undetectable in motoneurons^45^, but p75 persists at the neuromuscular synapse, where it is associated with motor terminals and postsynaptic AChR clusters, and has also been reported in Schwann cells^15^. At this stage, its absence impairs synaptic vesicle availability, delays postsynaptic maturation, and results in muscle weakness and reduced motor coordination^25^. However, whether p75 continues to play a role at the aged NMJ remained unknown. In this study, our immunohistochemical analyses revealed p75 immunoreactivity associated with motor axon terminals and postsynaptic AChR aggregates, supporting its potential role in the organisation and physiology of neuromuscular synapses during aging. Due to technical limitations, our experiments cannot rule out that p75 is expressed in other non-synaptic cell types, including tSCs or other glial/immune cells. Future research is needed to identify the precise cellular sources of p75 in aged extra-synaptic regions. Furthermore, because p75 is absent throughout the entire lifespan in this model, our data do not distinguish effects arising from its role during early NMJ development, including maturation and polyneuronal synapse elimination, and those reflecting a direct requirement in aging synapses. Thus, while our findings support a role for p75 in NMJ stability in aged animals, they do not isolate aging-specific mechanisms. Cell type specific and temporally controlled gene deletion approaches will be required to resolve these contributions.

At the NMJ, aging is characterised by several morphological changes, such as fragmentation, denervation, and the formation of axonal escape fibres^5,6^. Notably, neurotrophin signalling has been shown to regulate aging-related changes in NMJ morphology and physiology. Aged muscles show a significantly decreased expression of TrkB.t1, an endogenous truncated isoform of TrkB with essential roles in NMJ organisation^46^. Consistently, aged muscles display a marked reduction in the BDNF/TrkB signalling that potentiates NMJ neurotransmission in adult muscles^47–49^. However, the structural contribution of this pathway to NMJ organisation remains critical, as experimental disruption of BDNF/TrkB signalling induces presynaptic abnormalities and increased denervation even in early-aged mice (18-mo-old)^47^. In this context, it is also relevant that other neurotrophins support NMJ maintenance during ageing. For instance, NT3-based gene therapy exerts beneficial effects in aged muscle, which may reflect mechanisms dependent on p75, a receptor where multiple neurotrophin pathways converge^50^. Although recent findings indicate a ~ 50% reduction in p75 protein expression in EDL muscle extracts from aged rats^46^, our analysis shows that p75 mRNA levels are not significantly altered in NMJ-enriched fractions from diaphragm muscle between adulthood and aging, underscoring differences related to species, muscle type, and the level of analysis (protein versus transcript). Consistent with this, recent transcriptomic and single-nucleus studies have revealed substantial heterogeneity in gene expression programs across skeletal muscles and myonuclear subtypes, including NMJ-associated subsynaptic nuclei^51,52^. Moreover, independent studies have reported muscle-specific differences in p75 expression and regulation under physiological and pathological conditions^53,54^. In this context, the increased p75 mRNA expression detected in extrasynaptic regions of the aged diaphragm may relate to satellite cell–associated regenerative processes previously described in adult muscle^55,56^.

Although aged NMJs exhibit presynaptic molecular changes, such as reduced expression of the active forms of Munc18-1 and SNAP-25 of the SNARE-SM complex, as well as downregulation of P/Q-VGCC and muscarinic pathways^46^, we found that the absence of p75 did not affect presynaptic organisation at the NMJ. Except for a trend toward enlarged axonal diameter and reduced swelling, the occurrence rate of two parameters associated to muscle denervation, NMJ poly-innervation and axonal escape fibres, was not affected. Despite these preserved phenotypes, our findings showed a significant reduction in the proportion of presynaptic coverage of muscle postsynaptic domains in p75⁻/⁻ mice, indicating that the absence of p75 exacerbates NMJ denervation, a hallmark of muscle aging^57^. Partial denervation and altered neuromuscular activity are known to modulate the fetal γ-AChR subunit expression as well as neurotrophin signaling at the NMJ, respectively^42,43^. In aging and denervation models, changes in the balance of neurotrophins and their receptors have been reported, indicating that this signaling axis is responsive to synaptic stress^46,47^. Particularly, a significant aging-dependent change in the stoichiometry of the BDNF and NT-4 due to increased NT-4 has been reported^46^. In this context, our findings showing no detectable changes in AChR γ-subunit, BDNF, or TrkB levels in aged p75⁻/⁻ muscles are consistent with partial and heterogeneous denervation, which may destabilise NMJ structure and function without eliciting robust, global molecular reprogramming. Given the low abundance of γ-AChR in postnatal skeletal muscle, these results should be interpreted with caution, despite prior validation of the antibody under denervation conditions^34^. This pattern resembles that observed in aging and neurodegenerative contexts, where limited denervation is sufficient to impair neuromuscular function in the absence of evident myopathic or transcriptional signatures.

Interestingly, we also found a lower percentage of tSCs covering motor axon terminals, consistent with the aging phenotype of tSCs, in which they fail to fully cap the NMJ, suggesting that this physiological process is exacerbated in p75⁻/⁻ mice^7,58^. In adulthood, p75 expression in tSCs is required for synapse maintenance and to guide motor axons during reinnervation, a process characterised by increased p75 expression in these cells^7,31,59,60^. Similarly, p75 is expressed in Schwann cells and plays a role in motor axon myelination^31^. Therefore, it is plausible to speculate that tSC retraction in aged NMJs results from decreased p75 signalling, which contributes to the significant denervation observed in these animals.

Our findings indicate that the postsynaptic morphology of aged NMJs from p75⁻/⁻ muscles does not significantly differ from controls. This suggests that the delayed maturation and reduced morphological complexity previously observed in adult p75⁻/⁻ mice (4-6-mo-old)^25^ is somehow compensated upon aging. Only NMJs in the proximal LAL muscle, but not in the distal lumbrical muscle, display smaller perimeters in p75⁻/⁻ mice, correlating with the smaller pretzel perimeter observed in adult p75⁻/⁻ mice^25^. Although NMJ fragmentation is a prevalent feature of aging in murine species^6^, and inhibition or absence of TrkB or NT4 leads to postsynaptic fragmentation^61–63^, the absence of p75 did not alter this parameter. Notably, distal lumbrical muscles exhibit a higher percentage of NMJ postsynaptic domains with more fragments during aging compared to the proximal LAL muscle. In this regard, it is relevant to mention that aged muscles display a similar or even increased synaptic transmission^64^, and long-term reinnervated murine NMJs fully recover synaptic transmission despite persistent postsynaptic fragmentation^27^. Also, the NMJ of humans and other species exhibits fragmented postsynaptic domains^65,66^. Therefore, NMJ fragmentation may not solely indicate NMJ aging but could also represent an adaptive mechanism to maintain synaptic function.

Despite minor changes in overall postsynaptic morphology, our findings reveal that postsynaptic stability is significantly compromised in aged p75⁻/⁻ mice. Using two-colour BTX staining over a 7-day interval —based on the 14-day half-life of AChRs in the muscle membrane^45,67^— we observed reduced AChR half-life in aged p75⁻/⁻ mice, while the insertion of new AChRs into the muscle membrane was unaltered compared to aged controls. In adult mice, postsynaptic stability was preserved in p75⁻/⁻ mice^25^, suggesting that specific aging-associated mechanisms affect postsynaptic stability. Interestingly, a similar reduction in surface AChRs has been observed in animal models of NMJ decline, including prolonged high-fat-diet obesity^68^ neuromuscular diseases^69^, and denervated synapses^45,67,70^. Considering that AChR density did not vary between p75⁻/⁻ animals and controls, it is possible that endocytosed receptors are recycled back to the muscle surface at a lower rate than those targeted for degradation.

One of the most striking alterations we observed in aged p75⁻/⁻ mice is muscle weakness, a phenotype consistent with previous studies reporting similar deficits at comparable ages^71^. Indeed, muscle weakness is more pronounced in aged animals compared to adult p75⁻/⁻ mice^25^. In line with these findings, genetic deletion of the truncated TrkB variant TrkB.t1 has been shown to improve muscle strength^72^. In this context, it is relevant that p75 is expressed in developing muscle, particularly in muscle stem cells, where it promotes regeneration, differentiation, and myogenesis^56,71^. Indeed, cleavage of the intracellular domain of p75, which modulates gene expression, occurs in primary cultures of proliferating and differentiating myoblasts^54^. Although p75/proNGF signalling has been implicated in slow- to fast-twitch muscle fibre transition^54^, our results revealed no differences in fibre type composition in p75⁻/⁻ mice. Likewise, our findings show that the absence of p75 did not result in muscle fibrosis or degeneration/regeneration cycles, as previously reported in adult p75⁻/⁻ mice^25^. Only a trend to atrophy was observed in glycolytic-type fibres of aged p75⁻/⁻ mice. This pattern could be related to the critical role of BDNF–TrkB in maintaining of fast/glycolytic fibres^54,73^, representing a potential mechanism for the selective vulnerability of these fibres. In this context, NMJs are known to display muscle-specific morphological features that reflect differences in fibre-type composition and functional demands^39,74^. While this heterogeneity may influence individual parameters, the convergence of phenotypes across multiple muscles supports the conclusion that impaired NMJ stability is a general consequence of persistent p75 deficiency.

Beyond the NMJ, p75 has different functions in the central and peripheral nervous system. In the CNS, it is mostly linked to neuronal apoptosis and reduced plasticity, and inhibition of p75 has been proposed as a therapeutic strategy to limit neurodegeneration and promote recovery^75–77^. In contrast, in the PNS, p75 has more supportive roles. It contributes to the myelination of peripheral axons^31,78^, the development of sensory neurons^26^, and the stability of adult neuromuscular synapses^25^. The p75 receptor is also strongly up-regulated after nerve injury in motoneurons and Schwann cells, where it controls axonal regeneration, reinnervation, and myelination^79^. Importantly, in amyotrophic lateral sclerosis, a disease characterised by degeneration of the neuromuscular synapse, there is an imbalance in the expression of muscle neurotrophins and their receptors^80^. In this context, p75 decreases its expression in muscle tissue but increases in urine, and has been proposed as a marker of disease progression^80,81^.

Taken together, our findings support a model in which the pronounced reduction in muscle strength primarily reflects a dominant impact of persistent p75 deficiency on NMJ function rather than overt muscle degeneration. Maximal force output is highly sensitive to partial NMJ dysfunction, and in aging rodents NMJ instability correlates with declines in muscle strength independently of muscle mass loss^38^. Moreover, age-related decline preferentially affects fast, glycolytic fibres and NMJ integrity, a selective vulnerability associated with greater functional weakness in vivo^82^. In this context, the selective atrophy of glycolytic fibres in p75⁻/⁻ muscles, together with the absence of classical markers of chronic denervation or myopathy, supports NMJ destabilization as a key contributor to weakness during aging. In this regard, while inhibition of p75 may be beneficial in the CNS, therapeutic strategies must be considered with caution in the peripheral context, as they could potentially accelerate NMJ decline.

## Methods

### Animals

The C57BL/6 J strain B6.129S4-Ngfrtm1Jae/J p75NTR − /− mice^26^ and control littermate mice were obtained from The Jackson Laboratory (Sacramento, California, USA). Mice of both sexes were used, aged 24 to 26 months, representative of aging analyses. Additionally, 5 and 26-month-old C57BL/6 J mice were included in the study as controls. Mice were maintained under appropriate humidity, hygiene, light cycles, and temperature conditions, with *ad libitum* access to food and water (Prolab RMH-3000, LabDiet). Mice were euthanised via inhalatory isoflurane anesthesia overdose. Experimental procedures were approved by the Bioethics Committee at the University of Concepcion, Chile, and adhere to the guidelines imposed by the Bioethics Committee of the National Commission for Scientific and Technological Research, Chile (ANID), following the ethical standards outlined in the Animals (Scientific Procedures) Act 1986, UK.

### NMJ staining and analyses

Lumbrical and Levator auris longus (LAL) muscles were dissected and whole-mounted, fixed in 0.5% formaldehyde (FA) in 1X phosphate-buffered saline (PBS) at 22°C for 90 minutes. Samples were incubated with 0.1 M glycine in 1X PBS, permeabilised with PBST (1X PBS/0.5% Triton X-100) and blocked with 4% bovine serum albumin (BSA) dissolved in PBST for 12-16 hours at 4°C. A co-incubation with the Ig antigen-binding domain fragment was performed to decrease nonspecific staining. Muscles were then incubated with mouse monoclonal antibodies raised against neurofilament (2H3; 1:300; Developmental Studies Hybridoma Bank, DSHB, University of Iowa, USA; Catalog Nr: AB_531793) and synaptic vesicles (SV2) (1:50; DSHB; Catalog Nr: AB_2315387), along with an anti-S100 antibody (1:5; Dakocytomation, Santa Clara, CA, USA; Catalog Nr: IR504) or p75 (1:500; Cell Signaling Technology, Danvers, MA, USA; Catalog Nr: #8238) in 4% BSA-PBST for 30 minutes at room temperature and then overnight at 4°C. The tissues were subsequently incubated with the respective secondary antibodies (1:300; Jackson ImmunoResearch, West Grove, PA, USA) in 4% BSA-PBST containing Alexa488-conjugated α-bungarotoxin (BTX) (1:500; Invitrogen, Carlsbad, CA, USA; Catalog Nr: B13422) and DAPI (1:1000; Thermo Fisher, Waltham, MA, USA; Catalog Nr: D1306) for 12-16 hours at 4°C. Samples were washed in 1X PBS for 10 minutes at 22°C, flat-mounted between two coverslips, and imaged^27^. IMARIS^©^ software was used to generate 3D representations of the images. Cell volume was defined from confocal image stacks, and 3D surface reconstructions were performed using automatically optimized parameters.

### Quantitative analyses at the NMJ

To analyse NMJ morphology, z-stack images were collected at 1μm intervals using a Zeiss LSM 780 confocal microscope at the CMA Bio-Bio facility, University of Concepcion, Chile. Maximal intensity projection images were reconstructed in 3D using ImageJ software and analysed to determine the proportion of different postsynaptic NMJ morphologies. These were categorised into those with a single peripheral opening (still maturing) or those with multiple peripheral openings (mature pretzels). The morphology of 39-42 NMJs per mouse was manually determined and expressed as a percentage of the total. Images were obtained by processing confocal z-stack images using Imaris software. The fragmentation, area, perimeter, and diameter of >50 AChR densities per mouse were determined for each postsynaptic structure using ImageJ software, as described^39^. To determine NMJ innervation, the free-AChR presynaptic and the total AChR positive areas of >35 NMJs per mouse were calculated. Data are expressed as the fractional apposition between the pre- and postsynaptic domains. Based on previous reports, NMJs with a pre/postsynaptic apposition > 60% was considered innervated; in turn, a < 20% overlap was considered as denervation, while 20 – 60% apposition was classified as partial innervation^39^. To analyse AChR stability, two α-bungarotoxin (α-BTX) assays were performed, as described^34^. Briefly, mice anesthetised with isoflurane (2.5% v/v isoflurane mixture with 0.8-1 L/min oxygen) were subcutaneously injected over the LAL muscle with α-BTX conjugated to the Alexa-488 fluorophore (BTX-1) dissolved in PBS. Seven days later, mice were euthanised, and the LAL muscles were dissected out, fixed in 0.5% v/v formaldehyde at room temperature, washed, and stained with α-BTX conjugated to the Alexa-647 fluorophore (BTX-2) for 60 min at room temperature. A range of 38–63 NMJs per mouse were imaged using confocal microscopy, with acquisition parameters kept constant. The BTX-1-to-BTX-2 fluorescence intensity ratio was analysed using ImageJ^34^.

### Western blot

Total protein extracts from the Gastrocnemius muscles of aged p75⁻/⁻ and control mice were loaded into each lane and fractionated by sodium dodecyl sulfate-polyacrylamide gel electrophoresis (SDS-PAGE; 8-10%) and subsequently transferred onto polyvinylidene difluoride (PVDF) membranes (EMD Millipore Corp.). The membranes were probed with antibodies against atrogin-1 (1:1,000; ECM Biosciences, Versailles, KY, USA; Catalog Nr: AP2041), AChR γ-subunit (1:1000, Invitrogen; Catalog Nr: PA568078), BDNF (1:1000, Invitrogen; Catalog Nr: YL4141846), or TrkB (1:1000, BD Biosciences, San Jose, CA, USA; Catalog Nr: AB_397508). The AChR γ-subunit antibody has been previously validated in denervated skeletal muscle, where γ-AChR expression is robustly upregulated^34^. Anti-GAPDH antibodies (1:1,000; Santa Cruz Biotechnology, Dallas, TX, USA; Catalog Nr: SC-2357) were used as a loading control. All immunoreactions were visualised using enhanced chemiluminescence (Thermo Fisher Scientific, Waltham, MA, USA) and acquired with a FOTO/Analyst Luminary Workstations System (Fotodyne, Inc., Walnut Ridge, WI, USA). Data are expressed as the ratio atrogin-1 to GAPDH band intensities, which were quantified using ImageJ^25^.

### Analysis of muscle cryosections

Frozen TA muscles were embedded in optimal cutting temperature (OCT) compound (Sakura Fine Technical Co., Torrance, CA), sectioned every 20 µm using a cryostat (Thermo Scientific Microm HM 525), and mounted on slides coated with Vectabond (Vector Laboratories). The sarcolemma and nuclei were stained with Alexa488-conjugated wheat germ agglutinin (WGA; 1 µg/ml; Invitrogen; Catalog Nr: W11261) and 0.3 µM DAPI (Thermo Fisher), respectively. Cryosections were also stained with a solution of reduced NADH in Tris buffer, pH 7.4 (Sigma) for 45 minutes, and fibres were classified into NADH-TR+ Oxidative (dark blue), intermediate (blue), and NADH-Glycolitic fibres (light blue). The Feret diameter of >100 fibres per type per mouse was determined using ImageJ software and expressed as a percentage of the total. The potential presence of fibrosis was assessed by immunostaining for fibronectin (1:5000; Sigma-Aldrich) deposition and by second harmonic generation imaging to visualise collagen deposition using an inverted Zeiss LSM 780 multiphoton laser scanning confocal microscope at the CMA Bio-Bio facility (University of Concepcion, Chile), as described^25^.

### RNA isolation and RT-qPCR

To obtain the synaptic and extra-synaptic regions, diaphragms were dissected in stereomicroscope. Muscles were homogenised using sonicator (Misonix) in Trizol (Ambion) and RNA was extracted following the manufacturer’s instructions. cDNA synthesis from 1ug of RNA, was performed using the RevertAid Kit (Invitrogen). RT-qPCR was performed on a Rotor-Gene Q (Qiagen) using SsoAdvanced Universal SYBR Green Supermix (Bio-Rad) and primers targeting p75NTR and GAPDH. The reactions for RT-qPCR were performed using the following thermal cycler conditions: 95˚C for 3 min, 45 cycles of 95˚C for 20 s, 60˚C for 20 s and 72°C for 20 s to p75NTR and 95˚C for 3 min, 40 cycles of 95˚C for 20 s, 57˚C for 20 s and 72°C for 20 s to GAPDH. After PCR amplification, a melting curve analysis was performed to assess PCR specificity. Transcript levels were normalised to GAPDH transcript levels. Relative fold change in expression was calculated using the ΔΔCT method. All primers used in this study are listed in Table 1.

**Table 1:** Primers used for p75 expression analyses^84^

Gene	Forward sequence	Reverse sequence
p75NTR (NGFR)	CTAGGGGTGTCCTTTGGAGGT	CAGGGTTCACACACGGTCT
GAPDH	GAGTAAGAAACCCTGGACCAC	TCTGGGATGGAAATTGTGAGG

### Weight test

Mice were challenged to hold increasing weights (19, 24, 33, 40, 48, and 53 g). The final score is calculated by relating the animal’s body weight to the weight held and the duration for which the weight was carried^83^.

### Statistical analyses

Data was analysed using GraphPad Prism 8 and RStudio version 2025.9.0.387 software and presented as mean ± SEM. Statistical analyses were performed using Student’s t-test for single variables and two-way ANOVA for multiple variables. *P*-values below 0.05 were considered statistically significant, denoted by *, **, and ***, indicating *p* < 0.05, *p* < 0.01, and *p* < 0.001, respectively. In the figures, ‘*n*’ represents the number of NMJs per mouse, while ‘*N*’ represents the number of mice for each condition. Source data underlying these analyses are provided in Supplementary Data 1. A list of antibodies and other reagents used in these studies, including their source and catalog number, are provided in Supplementary Table 1.

## Supplementary information

Supplementary Figures S1-S4_npj Aging Manuscript Perez et al_20260322

Supplementary_Table 1_20260422

Supplementary_DATA1_20260322

## Data Availability

All data generated or analysed during this study are included in this published article and its Supplementary Information file (Supplementary Data 1).

## Code availability

No custom code was used in this study.
