# Supplementary material for: p75 neurotrophin receptor preserves neuromuscular synapse stability and muscle strength during aging
Source: NPJ Aging. 2026 May 16;12(1):105. doi: 10.1038/s41514-026-00399-1 (PMC13434739; doi:10.1038/s41514-026-00399-1)
Supplement: Supplementary file 1 [file 41514_2026_399_MOESM1_ESM.docx]

**This file includes Supplementary Figures for:**

p75 neurotrophin receptor preserves neuromuscular synapse stability and muscle strength during aging.

**Authors:**

Viviana Pérez^1,2^*, Sebastián Aedo-Cares^2^, Francisca Bermedo-García^2,3^, Jessica Mella^2,3^, Camila Uribe-Martínez^1^, Francisca C. Bronfman^4^*, Juan Pablo Henríquez^2,3^*.

**Affiliations:**

^1^Departamento de Fisiología, Facultad de Ciencias Biológicas, Universidad de Concepción, Concepción, Chile.

^2^Departamento de Biología Celular, Facultad de Ciencias Biológicas, Universidad de Concepción, Concepción, Chile.

^3^Neuromuscular Studies Lab (NeSt Lab), Instituto de Anatomía, Histología y Patología, Facultad de Medicina, Universidad Austral de Chile, Valdivia, Chile.

^4^Institute of Biomedical Sciences (ICB), Faculty of Medicine, and Faculty of Life Sciences, Universidad Andrés Bello, Santiago, Chile.

**Supplementary Figure S1. Uncropped and unprocessed blots corresponding to Figure 6c.**

Full-length blots for AChR γ-subunit (upper panel) and GAPDH (lower panel) are shown. Molecular weight (MW) markers are indicated on the left of each blot. Dashed-line squares highlight the regions used in the cropped images in Figure 6c. All scans were obtained directly from the original blots without contrast or gamma adjustments. For consistency, bands were inverted in Figure 6c (Ctrl on the left, p75⁻/⁻ on the right).

**Supplementary Figure S2. Uncropped and unprocessed blots corresponding to Figure 6d.**

Full-length blots for BDNF (upper panel) and GAPDH (lower panel) are shown. Molecular weight (MW) markers are indicated on the left of each blot. Dashed-line squares highlight the regions used in the cropped images in Figure 6d. All scans were obtained directly from the original blots without contrast or gamma adjustments. For consistency, bands were inverted in Figure 6d (Ctrl on the left, p75⁻/⁻ on the right).

**Supplementary Figure S3. Uncropped and unprocessed blots corresponding to Figure 6e.**

Full-length blots for TrkB (upper panel) and GAPDH (lower panel) are shown. Molecular weight (MW) markers are indicated on the left of each blot. Dashed-line squares highlight the regions used in the cropped images in Figure 6e. All scans were obtained directly from the original blots without contrast or gamma adjustments. For consistency, bands were inverted in Figure 6e (Ctrl on the left, p75⁻/⁻ on the right). Samples KO2 and KO5 (p75⁻/⁻) were excluded from quantification in Fig. 6e due to technical loading errors identified in the original blots.

**Supplementary Figure S4. Uncropped and unprocessed blots corresponding to Figure 7e.**

Full-length blots for Atrogin (upper panel) and GAPDH (lower panel) are shown. Molecular weight (MW) markers are indicated on the left of each blot. Dashed-line squares highlight the regions used in the cropped images in Figure 7e. All scans were obtained directly from the original blots without contrast or gamma adjustments.
