# Supplementary material for: p75 neurotrophin receptor preserves neuromuscular synapse stability and muscle strength during aging
Source: NPJ Aging. 2026 May 16;12(1):105. doi: 10.1038/s41514-026-00399-1 (PMC13434739; doi:10.1038/s41514-026-00399-1)
Supplement: Supplementary file 2 [file 41514_2026_399_MOESM2_ESM.docx]

**This file includes Supplementary Materials for:**

p75 neurotrophin receptor preserves neuromuscular synapse stability and muscle strength during aging.

**Authors:**

Viviana Pérez^1,2^*, Sebastián Aedo-Cares^2^, Francisca Bermedo-García^2,3^, Jessica Mella^2,3^, Camila Uribe-Martínez^1^, Francisca C. Bronfman^4^*, Juan Pablo Henríquez^2,3^*.

**Affiliations:**

^1^Departamento de Fisiología, Facultad de Ciencias Biológicas, Universidad de Concepción, Concepción, Chile.

^2^Departamento de Biología Celular, Facultad de Ciencias Biológicas, Universidad de Concepción, Concepción, Chile.

^3^Neuromuscular Studies Lab (NeSt Lab), Instituto de Anatomía, Histología y Patología, Facultad de Medicina, Universidad Austral de Chile, Valdivia, Chile.

^4^Institute of Biomedical Sciences (ICB), Faculty of Medicine, and Faculty of Life Sciences, Universidad Andrés Bello, Santiago, Chile.

| **Target** | **Antibody origin** | **Dilution/**  **concentration** | **Method** | **Catalog Number** | **Origin** |
| --- | --- | --- | --- | --- | --- |
| 2H3 | Mouse monoclonal | 1:300 | IHC | AB_531793 | Developmental Studies Hybridoma Bank, DSHB |
| SV2 | Mouse monoclonal | 1:50 | IHC | AB_2315387 | Developmental Studies Hybridoma Bank, DSHB |
| S100 | Rabbit Polyclonal | (1/5) | IHC | IR504 | Dakocytomation |
| GAPDH | Mouse monoclonal | 1:1000 | WB | SC-2357 | Santa Cruz |
| Atrogin-1 | Rabbit Polyclonal | 1:1000 | WB | AP2041 | ECM Biosciences |
| P75 | Rabbit polyclonal | 1:500  1:1000 | IHC  WB | #8238 | Cell Signaling |
| AChR γ-subunit | Rabbit polyclonal | 1:1000 | WB | PA568078 | Invitrogen |
| BDNF | Rabbit polyclonal | 1:1000 | WB | YL4141846 | Invitrogen |
| TrkB | Mouse monoclonal | 1:1000 | WB | AB_397508 | BD Biosciences |
| Secondary antibody | Rabbit polyclonal HRP | 1:5000 | WB | A9169 | Sigma-Aldrich |
| Secondary antibody | Mouse monoclonal  HRP | 1:5000 | WB | A44146 | Sigma-Aldrich |
| Cy3 anti-mouse | Donkey polyclonal | 1:300 | IHC | 715-165-150 | Jackson ImmunoResearch |
| Cy2 anti-rabbit | Donkey polyclonal | 1:300 | IHC | 711-225-152 | Jackson ImmunoResearch |
| **Other reagents** |  | | | | |
| DAPI | -- | 300nM | IHC | D1306 | Thermo Fisher |
| Alexa488- wheat germ agglutinin (WGA) | -- | 1µg/ml | IHC | W11261 | Invitrogen |
| BTX- Alexa488 | -- | 1:500 | IHC | B13422 | Invitrogen |
| BTX-1 Alexa-488 | -- | 4 μg/ml | AChR  Dynamics | B13422 | Invitrogen |
| BTX-2 Alexa-647 | -- | 2 μg/ml | AChR  Dynamics | B35450 | Invitrogen |

**Supplementary Table 1. Antibodies and key reagents used in this study.**

The table shows the origin, dilution/concentration, the method(s) in which they were used, catalog number, and origin of antibodies and key reagents used within these studies. IHC: Immunohistochemistry, WB: Western Blot.
